# Pleiotropy across academic subjects at the end of compulsory education

**DOI:** 10.1038/srep11713

**Published:** 2015-07-23

**Authors:** Kaili Rimfeld, Yulia Kovas, Philip S. Dale, Robert Plomin

**Affiliations:** 1 King’s College London, MRC Social, Genetic and Developmental Psychiatry Centre, Institute of Psychiatry, Psychology & Neuroscience, London, SE5 8AF, UK; 2 Goldsmiths, University of London, Department of Psychology, London, SE14 6NW, UK; 3 Tomsk State University, Laboratory for Cognitive Investigations and Behavioural Genetics, Tomsk, 634050, Russia; 4 University of New Mexico, Department of Speech and Hearing Sciences, Albuquerque, NM, 87131, USA

## Abstract

Research has shown that genes play an important role in educational achievement. A key question is the extent to which the same genes affect different academic subjects before and after controlling for general intelligence. The present study investigated genetic and environmental influences on, and links between, the various subjects of the age-16 UK-wide standardized GCSE (General Certificate of Secondary Education) examination results for 12,632 twins. Using the twin method that compares identical and non-identical twins, we found that all GCSE subjects were substantially heritable, and that various academic subjects correlated substantially both phenotypically and genetically, even after controlling for intelligence. Further evidence for pleiotropy in academic achievement was found using a method based directly on DNA from unrelated individuals. We conclude that performance differences for all subjects are highly heritable at the end of compulsory education and that many of the same genes affect different subjects independent of intelligence.

Academic achievement at the end of compulsory education is of major societal interest and is critical for students because the exam results play a substantial role in making decisions about further education and employment. Furthermore, educational achievement has been shown to be an independent predictor of many life outcomes, including career success, health and even life expectancy^1^,^2^. It is reasonable to assume that schools have a major effect on educational achievement, because children have to be taught, e.g., how to read and how to solve mathematical problems; however, children differ in their educational achievement within the same school and even the same classroom, indicating that factors other than school differences must be involved in individual differences in achievement^3^,^4^. Twin studies have shown that educational achievement is highly heritable in early and middle school years; that is, individual differences in academic achievement are to a large extent (around 60%) explained by inherited differences in their DNA sequence^5^,^6^,^7^,^8^,^9^,^10^,^11^,^12^,^13^. The heritability for academic achievement in core subjects is also substantial at the end of compulsory education in the UK^14^.

A key unresolved question raised by the results of previous studies is the genetic architecture underlying the heritability of academic subjects. Do the same or specific genetic factors influence the wide range of academic subjects taught at school? Previous research has used multivariate genetic analysis to study the shared aetiology between different academic subjects. Multivariate genetic analysis estimates the genetic contribution to the phenotypic correlation between traits and derives the genetic correlation, which corresponds to the correlation between genes that affect the two traits, independent of the heritabilities of the traits; the genetic correlation is an index of pleiotropy (the multiple effects of genes)^3^. Previous multivariate genetic studies, which have been limited to the core academic subjects of English, mathematics, and science at early stages of schooling^15^, have reported substantial genetic correlations between core academic subjects^13^,^15^,^16^,^17^,^18^. Further evidence for pleiotropy between core subjects was provided in a recent study, which reported a substantial genetic correlation between English and mathematics at age 12, using both the twin design and an analysis based on children’s DNA^19^. To our knowledge, no multivariate study has been conducted investigating the genetic architecture at the end of compulsory education for the wider range of academic subjects taught at schools, which is the purpose of the present study.

What drives the heritability of achievement in such different academic subjects and pleiotropy between subjects? The strongest predictor of general educational achievement is general cognitive ability (intelligence)^20^,^21^. Intelligence, just as academic achievement, is highly heritable, as demonstrated by quantitative genetic studies, with heritability estimates consistently indicating that about half of the variance (.50) is explained by genetic factors^22^. Research suggests that genetic factors substantially mediate the links between intelligence and academic achievement in core subjects in early school years^13^,^20^,^23^. These findings have led to the Generalist Genes Hypothesis, which posits that the same genes largely affect a wide range of cognitive and learning abilities^15^,^24^. Further evidence for the Generalist Genes Hypothesis comes from studies using the DNA of unrelated individuals in Genome-Wide Complex Trait Analysis^25^ (GCTA, a method also used in the present study), where the genetic correlations between reading, mathematics and first language achievement and general cognitive ability were highly similar to twin study estimates, with an average genetic correlation of .70^26^. For these reasons, intelligence is a likely candidate contributing to pleiotropy among academic subjects.

Research to date has focused on achievement in the core subjects of language, mathematics and science, subjects that seem to be more closely related to intelligence in the sense that they go beyond learning specific content, as compared to history and geography that involve more factual knowledge. This suggests the possibility that intelligence might differentially contribute to pleiotropy between particular core subjects. A more general related issue is whether pleiotropy extends beyond the core subjects, because studies to date have not considered whether there is genetic overlap between achievement in other academic subjects, such as history, geography, and art. No studies have investigated either of these issues – the extent to which pleiotropy exists beyond core subjects or the extent to which intelligence mediates these pleiotropic effects.

## The current study

The present study uses a large twin sample to investigate the genetic architecture of achievement across a wide range of standardized examination results at the end of compulsory education in the UK, ranging from core academic subjects such as mathematics, English and sciences, to art, humanities and second language learning. Importantly, this is the first study to assess the extent of shared genetic aetiology between GCSE exam grades and also the extent to which this pleiotropy is mediated by intelligence. Based on previous research at earlier ages, we expected to find substantial pleiotropy between the achievement grades for core academic subjects and hypothesized that this association extends to a wider range of academic subjects. We also hypothesized that intelligence is substantially responsible for this pleiotropy.

In addition to twin analyses, we used GCTA to test the same hypotheses using DNA alone for unrelated individuals. In our twin sample, one twin per pair was randomly selected to create a sample of unrelated individuals. GCTA was used to estimate genetic influence that can be explained by the combined additive effects of many common single nucleotide polymorphisms (SNPs) of academic subjects and the genetic correlation between them; we also assessed genetic overlap independent of intelligence. The combination of these two very different genetic designs (twins and GCTA) provides a powerful approach to investigate pleiotropy between a wide range of academic subjects at the end of compulsory education in the UK, with and without controlling for intelligence.

## Results

Means and standard deviations are presented in Table 1 by sex and zygosity for five groups: MZ males, MZ females, DZ males, DZ females, DZ opposite-sex pairs. ANOVA results show that the sex, zygosity and their interaction explain around 1% of the variance in achievement measures on average. For subsequent analyses, sex and age effects were removed using the regression method and data were transformed using the rank-based van der Waerden transformation, as explained in the Methods section. We have previously reported the full sex-limitation modeling results, which found little evidence for sex differences in genetic or environmental effects^14^; therefore, to increase power in the present analyses, the full sample, including opposite-sex twin pairs, was used.

### Twin analyses

**Figure 1:**
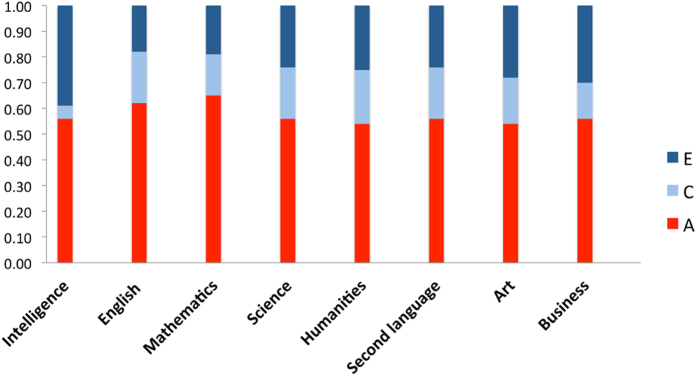
Univariate model-fitting results. A = additive genetic, C = shared environmental, E = non-shared environmental components of variance for GCSE exam grades and intelligence.

All GCSEs were highly heritable, demonstrating that genes explain a larger proportion of the individual differences (54–65%) than shared environmental factors, such as home and school environment combined (14–21%), as illustrated in Fig. 1. Intelligence at 16 also demonstrates substantial heritability (56%), with negligible effect of shared environmental influences (5%). (Twin intraclass correlations and full model fit statistics with confidence intervals are shown in Supplementary Table S1.)

Results of the multivariate genetic analyses indicated that all GCSE grades correlate substantially with intelligence phenotypically (.36–.56) and genetically (.44–.69). However, the correlations among academic achievement measures was even higher phenotypically (.49–.77) and genetically (.51–88) than with intelligence, as shown in Table 2.

**Figure 2:**
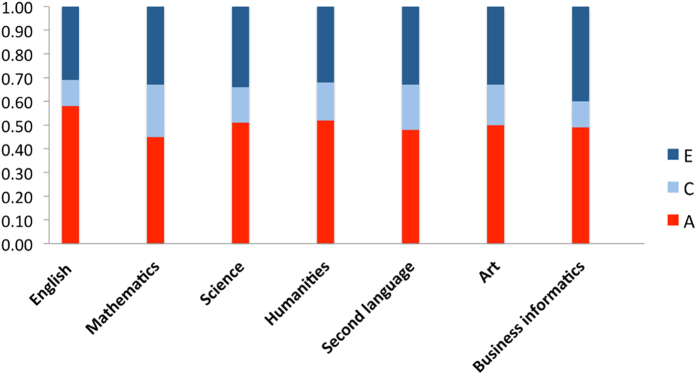
Univariate model-fitting results with GCSE exam grades corrected for intelligence. A = additive genetic, C = shared environmental, E = non-shared environmental components of variance.

Next, we removed the effect of intelligence from the GCSE exam grades using the regression method. After removing the effect of intelligence from the exam grades, the heritability of the achievement measures did not change much, ranging from 45–58%. The additive genetic (A), shared environmental (C) and non-shared environmental (E) proportions of variance for GCSE exam scores, independent of intelligence, are shown in Fig. 2. (Twin intraclass correlations and full model fit statistics with confidence intervals are listed in Supplementary Table S2.) Importantly, the heritability estimates for GCSE independent of intelligence are highly similar to the estimates uncorrected for intelligence, and although not a formal test of significance, the overlapping confidence intervals of the estimates provide further evidence for highly similar aetiology for GCSE results with and without controlling for intelligence (see Supplementary Tables S1 and S2). GCSE mathematics is an exception in that its heritability estimate of .65 dropped to .45 when intelligence was regressed out, suggesting that intelligence may play a stronger role in the heritability of mathematics performance.

Multivariate genetic analyses also indicated that the association between GCSE scores did not change substantially phenotypically or genetically after removing the effect of intelligence. As shown in Table 3, phenotypic correlations were substantial between a wide range of GCSE results independent of intelligence (.38–.69), as were genetic correlations (.49–81). The wide range of shared environmental correlations independent of intelligence (.01–.92) indicates that shared environmental influences vary between different subjects. The shared environmental correlations between core academic subjects of English, mathematics and science were substantial (.66–92), indicating that to a large extent the same shared environmental factors explain individual differences in these subjects, although the overall effect of shared environment was modest, accounting for about 20% of the variance. A possible exception is GCSE art, which seems influenced by different shared environmental factors compared to core academic subjects when the variance of intelligence is removed from exam grades.

An alternative, mathematically equivalent, way of investigating the extent to which intelligence mediates pleiotropy between GCSE subjects is to use multivariate Cholesky decomposition. Entering intelligence as the first variable in the model tests (i) the genetic overlap between intelligence and GCSE subjects, and (ii) the extent to which genetic overlap between GCSE subjects remains after controlling for intelligence. The results of Cholesky decomposition confirm the results of the correlated factor solution (Tables 2 and 3), as indicated by the standardized residual paths estimates presented in Supplementary Table S3. The paths for the first latent variable (A1 in Supplementary Table S3) with intelligence entered as the first variable indicate that intelligence is significantly related genetically with all of the GCSE subjects, which reflects the substantial genetic correlations between intelligence and GCSE subjects seen in Table 2. However, the paths for the second latent variable (A2 in Supplementary Table S3), indicate that, independent of intelligence, significant genetic pleiotropy remains for all GCSE subjects. The subsequent latent variables show that significant genetic specificity also exists for all GCSE subjects beyond the pleiotropy shared with intelligence and beyond the pleiotropy shared with other GCSE subjects independent of intelligence. The order of the variables entered into the Cholesky decomposition can affect the results; therefore, we conducted the analyses by changing the order of the academic achievement measures in the analyses, but the results remained the same.

### GCTA

Multivariate GCTA analysis is currently limited to the bivariate case, therefore, we focused on the exam results of compulsory core subjects of English, mathematics and science as the exam grades of the compulsory subjects also provided us with the largest sample size. GCTA was conducted using those participants who had GCSE grades for core subjects (English, mathematics and science) as well as genome-wide genotype data. Table 4 presents the results of bivariate GCTA analyses, investigating the genetic relationship between core subjects, before and after regressing out intelligence. GCTA heritability (heritability due to the additive combined effects of common SNPs) was .21 for GCSE mathematics, .15 for GCSE English and .17 for GCSE science, although these estimates did not reach significance because of the limited power, as indicated by their large standard errors. These GCTA heritability estimates are lower than the heritability estimates calculated with the twin method because GCTA relies on additive genetic influence and variants tagged by the common SNPs genotyped on current DNA arrays. The genetic correlations between the grades for core academic subjects were near unity.

GCTAs were repeated with GCSE mathematics, GCSE English and GCSE science grades after removing the effect of intelligence. GCTA heritability independent of intelligence was .14 for GCSE mathematics, .13 for GCSE English and .11 for GCSE science. The genetic correlation remained near unity, indicating that the same SNPs explain the genetic variance in all three core academic subjects, independent of intelligence.

## Discussion

Our results demonstrate that educational achievement across a wide range of academic subjects from traditional core subjects of English, mathematics and science, to humanities, second language learning, business informatics and art at the end of compulsory education in the UK is highly heritable, with over half of the variance in children’s educational achievement explained by inherited differences in their DNA, rather than school, family and other environmental influences. These results are in line with our previous research at earlier school years and with results reported for core GCSE subjects^13^,^14^. The slight difference in heritability estimates in core GCSE subjects results from including opposite sex twin pairs in the sample, which were not included in our previous study, resulting in more conservative heritability estimates^14^. We have also demonstrated that this high heritability is not explained by intelligence alone, as the heritability remained high even after removing intelligence from the GCSE grades. This is consistent with our recent study that showed that high heritability of educational achievement is explained by many genetically influenced traits, not just intelligence^20^.

In the most novel contribution of the present study, we showed that academic subjects at the end of compulsory education in the UK are to a large extent influenced by the same genes, even when intelligence is controlled. The genetic correlation between various academic achievement measures was substantial (.51–.88) and this includes traditional academic subjects of English, mathematics and science as well as art and language learning. The genetic overlap between GCSE scores and intelligence at age 16 was also substantial (.44–.69); however, genetic correlations were higher between GCSE scores than between GCSE scores and intelligence. Despite the genetic overlap between GCSE scores and intelligence, an intriguing finding is that pleiotropy among academic subjects is to a large extent independent of intelligence, as the genetic correlations were still substantial even after statistically removing intelligence from the GCSE scores (.49–.81). The results were also confirmed using Cholesky decomposition of the same multivariate genetic analyses in which intelligence was included as the first variable in the model.

We have previously shown that heritability of educational achievement at the end of compulsory education (mean of English, mathematics and science GCSEs) is influenced by a range of cognitive and non-cognitive factors, not just intelligence^20^. The present study showed that even after intelligence has been controlled for, the academic subjects still correlate substantially both phenotypically and genetically. It is possible that the genetic mechanisms responsible for these associations are also influenced by many genetically influenced traits, such as personality, motivation, and psychopathology.

Further evidence for the strong pleiotropy in academic achievement at age 16 was provided by the GCTA results. Genetic influence on the core subjects of English, mathematics and science was shown by the amount of variance explained by the genome-wide SNPs of unrelated individuals. This GCTA heritability estimate is lower than the heritability estimate calculated with the twin method because GCTA relies on additive genetic influence and variants tagged by the common SNPs genotyped on current DNA arrays. However, our index of pleiotropy, the genetic correlation, is not biased, for reasons explained elsewhere^26^. Similar to the twin results, the GCTA genetic correlations between GCSE English, mathematics and science were near unity, and after regressing out intelligence, the genetic correlations remained close to unity. These results indicate that the same SNPs influence academic achievement grades in core subjects, independent of intelligence.

We could not mirror the full multivariate analyses we conducted using the twin design because GCTA analysis is so far limited to the bivariate case. As described in the method section, GCTA needs large samples to detect SNP heritability, and even larger sample sizes are necessary for bivariate GCTA analyses. The results reported here have large standard errors, which is a limitation of the study. The results show, however, that even with limited power, we were able to detect significant GCTA genetic correlations between English, mathematics and science.

This is the first large-scale twin study that used standardized examination grades for a wide range of academic subjects from mathematics to art. The GCSE exams are standardized and blind-graded, so are arguably a better measure of achievement than teacher ratings. Even though teacher ratings are based on more evidence than single exam grades, the estimates could be biased, especially during primary school education, as students are often taught by the same teacher in all classes during the early years of education.

The strong genetic influence seen here across a wide range of academic subjects and the high genetic correlations between all these subjects – especially after controlling for intelligence – is intriguing. It is possible that the strong genetic influence compared to the modest effects of shared family and school environments on academic achievement occurs because of the standardized curriculum in the UK. Environmental differences may be reduced in the UK because of its national standardized curriculum; heritability estimates could be high precisely because environmental differences are attenuated. In fact, it has been proposed that the heritability of educational achievement could be viewed as an index of equal educational opportunities^4^. Empirical evidence provides some support for this hypothesis as the heritability of educational attainment has been reported to be higher and shared environmental influences lower in a centralized educational system as compared to a decentralized educational system, such as the United States^27^.

We have demonstrated here that the high heritability of educational achievement extends from primary education to the end of compulsory education. This might be due to teachers preparing children for standardized UK-wide exams. Given the importance of GCSE grades for both schools and individuals, the preparation for exams is done in a relatively standardized way; therefore, the impact of environment on GCSE variance is reduced, which would increase the heritability estimate.

The high heritability could also be explained in terms of gene-environment correlation. Genotype-environment correlation is increasingly important during adolescent development, as children select, modify and evoke their experiences partly based on their genetic propensities. Gene-environment correlation does not only happen passively as children inherit both genes and home environment from their parents^4^. Notably, genes also affect the environment children choose; thus influencing both the aptitude and appetite for learning^14^.

The present study is the first multivariate research to demonstrate pleiotropy across a wide range of academic subjects at the end of compulsory education, using both the classic twin method and the new quantitative genetic method, GCTA. This evidence for the highly pleiotropic nature of achievement in academic subjects and intelligence goes against the belief of specific learning abilities, such as mathematics ability versus ability in language. It is important to note, however, that not all genetic effects are pleiotropic: the results also indicate specific genetic influence as the twin study estimates of genetic correlations are less than 1.0. Additionally, the shared environmental correlations show substantial overlap between academic subjects as well as some specificity, and the non-shared environmental influences between academic subjects are largely not overlapping. Discrepancies between exam grades at age 16 can be used to explore the distinct genetic and environmental factors involved in different areas of academic achievement. Identifying these subject-specific effects could lead to better understanding of possible candidates of early intervention for specific learning disabilities.

Limitations of our study begin with the standard assumptions of the twin design, which are described in detail elsewhere^3^,^28^. Another limitation involves assortative mating in which mate selection is based on trait similarity rather than at random. While assortative mating for personality measures is typically low (~.10), assortative mating on intelligence, especially on verbal ability, is substantial (~.40)^3^,^29^. Assortative mating could decrease the heritability estimates provided by the twin design as it would increase the DZ twin correlations relative to MZ twin correlations^3^,^29^. Because of these assumptions and limitations of the twin design, corroboration of the twin results using a completely different method based on DNA alone is important. Here we show that both the twin and GCTA methods yield extremely high estimates of genetic correlations among academic subjects, even when intelligence is controlled. Another possible limitation is the intelligence measure used in the twin analyses. Although verbal and non-verbal abilities capture major components of intelligence, there could be aspects of general cognitive ability that are not assessed by our index and could contribute to the genetic covariance between the range of exam grades at the end of compulsory education. It is notable, though, that the GCTA results yield similar results to those of twin analyses even though the twin analyses used different measures to index intelligence, including general knowledge and spatial ability tests. The similarity of results provides further evidence for genetic pleiotropy across GCSE subjects independent of intelligence.

Achievement at the end of compulsory education is of major societal and individual importance because results of the GCSE exams are used as a basis when making decisions regarding further education and employment. We have demonstrated here that genetic factors explain a large proportion of individual differences in academic achievement at the end of compulsory education in the UK. Our results also indicate that, to a large extent, the same genes influence achievement across a wide range of academic subjects, even when controlling for intelligence. When genes are found to be associated with one learning ability the same genes would likely be associated with other learning abilities. The results of this study could lead to new multivariate molecular genetic research that aims to identify genes responsible for the pleiotropy across academic subjects. Understanding the specific genetic and unique environmental factors influencing individual differences in educational achievement, and the complex interplay between them, could help educationalists develop effective personalized learning programs, so that every child could reach their maximum potential by the end of compulsory education.

## Methods

### Participants

The Twin Early Development Study (TEDS) sample was used. TEDS is a longitudinal study that has recruited over 16,000 twin pairs born in England and Wales between 1994–1996. Currently, more than 10,000 twin pairs remain actively involved in the study. The sample is a representative sample of the UK when compared to the UK National Statistics. Rich behavioural and cognitive data have been collected over many years from the participants, including measures of academic achievement. The twins have now completed compulsory education and are moving on to further education or the workforce^13^,^30^.

We included all twins with available GCSE achievement measures in the analyses. Participants with major medical or psychiatric conditions were removed from the analyses, as were those individuals with severe perinatal complications. Additionally, we excluded all twins who did not have English as their first language. Zygosity was assessed by the parent-questionnaire of physical similarity. This measure has previously been shown to be highly reliable (95%)^31^. DNA testing was conducted where zygosity was unclear from this questionnaire. The present study employed all individuals with available GCSE grades comprising an overall sample of 6,316 twin pairs (12,632 individuals): 2,245 monozygotic (MZ) twin pairs, 2,069 dizygotic (DZ) same-sex twin pairs and 2,002 opposite-sex twin pairs.

DNA has been genotyped for subsample of TEDS twins (one twin per pair), see *Genotyping*. Genome-wide genotypes were available for 3,152 individuals, which were matched to those participants with available grades for GCSE mathematics, GCSE English and GCSE science. This led to a sample of 2,572 unrelated individuals with GCSE mathematics grades, 2,601 individuals with GCSE English grades and 2,381 individuals with GCSE science grades; this sample was used to conduct the GCTA analyses. As intelligence scores were not available for all individuals, after correcting the GCSE grades for intelligence, the sample comprised 2,526, 2,561 and 2,345 individuals respectively.

### Measures

General Certificate of Secondary Education (GCSE) grades were used. GCSE is a standardized examination taken at the end of compulsory education in the UK. Children typically start GCSE courses at the age of 14 and the exams are taken at the age of 16. Students can choose from a variety of different courses such as mathematics, science, history, music, physical education, and modern foreign languages. English, mathematics and science are compulsory subjects. Some schools also require students to take one GCSE in a second language. Importantly, the subjects that students choose and their performance in the GCSE exams have profound impact on their further education and employment. The exams are graded from A* to G, with a U grade given for failed exams. Grades were coded from 11(A*) to 4(G) to have equivalent numerical comparisons. No information about the failed courses was available. Most pupils receive 5 or more grades between A* and C, which is the requirement for further education in the UK. GCSE grades were collected from parents or the twins themselves via questionnaires sent by mail or over the telephone. For 7,367 twins the grades were verified using the National Pupil Database (NPD; https://www.gov.uk/government/uploads/system/uploads/attachment_data/file/251184/SFR40_2013_FINALv2.pdf), yielding a correlation of 0.99 for mathematics, 0.98 for English and >0.95 for all the sciences.

We created composite measures for English (mean of English language and English literature grades), science (mean of single or double-weighted science, or, when taken separately chemistry, physics and biology grade), second language achievement (mean of any second language grade available), humanities (mean of history, religious studies, geography and media studies), business informatics (mean of statistics, business studies and ICT - information and communication technology), and art (mean of art, drama and music grades). Additionally, we used the GCSE grade for mathematics.

Intelligence was assessed at age 16 using verbal and non-verbal abilities administered online. Verbal ability was measured by Mill Hill Vocabulary test, a multiple-choice test^32^. Twins were presented with the target word on a computer screen and they had to choose a word that was closest in meaning to the target words. Non-verbal ability was measured by Ravens Progressive Matrices^33^, collected from the twins via internet at the age of 16. Twins were presented with incomplete patterns (‘matrices’) and were asked to identify the missing part to complete the pattern. Intelligence (general cognitive ability) was indexed by taking the mean of verbal and non-verbal abilities.

Because not all the TEDS birth cohorts participated in the intelligence assessment at 16, and not all of those had been genotyped, the sample with all relevant measures would not have been sufficient for the GCTA analysis of grades corrected for intelligence. For that reason, we chose to construct a composite measure of ‘g‘ using all earlier measures from the longitudinal study. A composite was used to assess intelligence in order to control for intelligence in GCSE mathematics, GCSE English and GCSE science: a robust measure of ‘g’ derived from intelligence data collected longitudinally across nine ages from early childhood to age 16. At age 2, mean ‘g’ measure was calculated as a mean of a parent-administered design drawing task^34^, a matching task (match to design)^35^, a brick building task, a folding task and a copy task^36^,^37^,^38^,^39^,^40^; at age 3, mean ‘g’ was calculated as a mean of a parent-administered oddity task (odd-one-out)^35^, a design drawing task^34^ , a matching task^35^, and a parent-reported conceptual knowledge task^36^,^37^,^39^; at age 4, ‘g’ was calculated as a mean of parent- administered oddity task (an odd one out task)^35^, a design drawing task, a draw a man task^34^, and a puzzle task^33^,^38^ ; at age 7, ‘g’ was calculated as a mean of conceptual grouping^34^ , a WISC similarities test^41^, a WISC vocabulary test^41^, and a WISC picture completion test^41^ all collected over telephone testing; age 9, ‘g’ was calculated as a mean of a shapes test^42^, a WISC vocabulary test^43^, a WISC general knowledge task^43^, and a puzzle test^42^ all collected by the booklets sent to the twins by post; age 10, ‘g’ measure was calculated as a mean of the Ravens standard Progressive Matrices^33^, a WISC vocabulary^43^, WISC picture completion^41^, and a WISC general knowledge test^43^ all collected via internet testing; at age 12, ‘g’ was calculated as a mean of the Ravens Progressive Matrices^33^, a WISC picture completion^41^, a WISC vocabulary^43^, and a WISC general knowledge test^43^ all collected via internet testing; at age 14, ‘g’ was computed as a mean of the Raven’s Progressive Matrices^33^ and a WISC vocabulary^43^; and age 16, ‘g’ was measured as described above. The mean score of intelligence was calculated across the nine ages.

Prior to any genetic analyses all measures were corrected for small age and sex differences (see Table 1), using the regression method, which is a standard practice in twin analyses. Standardized residuals of the variables were used in all further analyses. This method avoids overestimation of shared environmental influences, as twins are identical for age, and MZ twins are also identical for sex^44^. All outliers beyond three standard deviations from the mean were also removed from the analyses. The GCSE grades also showed negative skew, indicating a ceiling effect. Similar ceiling effect is observed in the UK population as demonstrated in the data from the National Statistics (NPD; https://www.gov.uk/government/uploads/system/uploads/attachment_data/file/251184/SFR40_2013_FINALv2.pdf). To correct for the ceiling effect, all measures were transformed to the standard normal distribution using the rank-based van der Waerden transformation^45^,^46^.

### Analyses

The measures were described in terms of means and variance, comparing boys and girls and identical and non-identical twins; mean differences for age and sex and their interaction were tested using univariate analysis of variance (ANOVA).

### Twin Design

The twin method was used to conduct univariate and multivariate genetic analyses. The twin method can be used to estimate the relative contribution of additive genetic (A), shared environmental (C) and non-shared environmental (E) effects on the variance and covariance of academic achievement measures and intelligence, by comparing monozygotic (MZ) correlations to dizygotic (DZ) correlations. MZ twins share 100% of their segregating genes, while DZ twins share around 50% of the segregating genes, just like any other siblings. Both MZ and DZ twin pairs are assumed to share 100% of their shared environmental influences, when growing up in the same family. Non-shared environmental influences are assumed to be unique to individuals, that is, uncorrelated between twins and not contributing to similarities between them. Heritability can be roughly calculated by doubling the difference between MZ and DZ correlations; shared environmental influences can be calculated by deducting the heritability estimate from the MZ correlations; and non-shared environmental influences can be calculated by deducting the MZ correlation from unity (following the Falconer’s formula)^28^. These parameters can be estimated more accurately, including calculating the confidence intervals, using structural equation modeling with maximum likelihood estimation, which also provides estimates for the model fit. Structural equation modeling program OpenMx was used for all model fitting analyses^47^.

Multivariate genetic analysis is the extension of univariate genetic analysis. While univariate twin analysis investigates the variance of one trait, multivariate genetic analysis investigates the genetic and environmental nature of covariance between multiple traits. Multivariate genetic analyses is a method that compares the MZ and DZ cross-twin cross-trait correlations to decompose the covariance between two or more traits of interest into additive genetic (A), shared environmental (C) and non-shared environmental (C) components^3^,^28^. As shown in Supplementary Fig. S1 for the correlated factor solution, the genetic correlation (*r*_G_) assesses the extent to which the same genes influence two traits; the shared environmental correlation (*r*_C_) indicates the extent to which the same shared environmental influences that make twins more similar on trait one, also make the twins more similar on trait two; and non-shared environmental correlations (*r*_E_), indicating the extent to which the same non-shared factors influence two traits. Importantly, genetic correlation is different from bivariate heritability estimate, as it does not take into account the heritability of two traits, which means that trait one and trait two can have low heritabilities, but the genetic correlation could be high, implying that if a gene were found for one trait, there would be a good chance that this gene would also be associated with trait two^3^. Alternative representation of the same analyses is the Cholesky decomposition (see Supplementary Fig. S1). The central question of Cholesky decomposition is the extent to which the heritability of trait one can be accounted for by the heritability of the other trait, thus answering the question ‘to what extent does the heritability of one variable explain the heritability of the other variables’. In the multivariate model, when studying how much variance in trait three is accounted for by trait two, the model controls for the variance of trait one. The Cholesky decomposition is conceptually similar to hierarchical regression, therefore, the order of the variables entered influences the results. Each variable in the model controls for the variance in the previous variable, as illustrated in Supplementary Fig. S1.

### Genome-Wide Complex Trait Analysis (GCTA)

GCTA is a technique that estimates genetic and residual components of variance directly from the DNA of unrelated individuals, unlike twin analysis that relies on family resemblance data^3^. In order to create a sample of unrelated individuals, we randomly selected one twin per pair for GCTA analyses. Studies to date have shown that the heritability of complex behaviours, such as academic achievement, is highly polygenic, influenced by a large number of genes, each having only a small effect^25^. This explains the relatively slow progress in identifying the specific genes involved in educational achievement, as well as most other traits in the life sciences. GCTA can estimate heritability directly from DNA while not identifying the specific genes involved. The GCTA method uses hundreds of thousands of SNPs (single nucleotide polymorphisms) from thousands of individuals to calculate the proportion of phenotypic variance due to the additive effects of common SNPs^3^. First, the genetic relatedness matrix (GRM) is calculated by weighing the pairwise genetic similarities with the allele frequencies across all SNPs on the array. All participants who are found to be even remotely related (genetic relatedness 0.025 or greater) are removed from the analyses, as this would otherwise bias the results^48^. The matrix of pair-by-pair genetic similarity of all the participants is compared to the matrix of their phenotypic similarity using residual maximum likelihood estimation, without testing the association of any single SNP individually^25^,^49^,^50^. The advantage of this method is that heritability estimates can be calculated using a sample of unrelated individuals; the disadvantage is that very large pools of participants are needed to detect overall genetic similarity from the matrix of hundreds of thousands of SNPs. Notably, the heritability estimate calculated using the GCTA method only assesses additive genetic effects, not gene-gene or gene-environment interactions and only common SNPs are analysed^50^. Univariate GCTA analyses can be extended to bivariate analyses by comparing the phenotypic covariance matrix to the GRM^51^,^52^. Prior to the GCTA analyses we adjusted the GCSE English, GCSE mathematics and GCSE science grades for age and sex, using the regression method. Additionally, to control for population stratification, the principal component analysis was conducted for 100,000 quality-controlled SNPs, and eight axes were identified with p < 0.05 using the Tracy Wisdom test; these eight principal components were added as covariates in the bivariate GCTA analyses^53^.

Power was calculated using an online tool for calculating power for GCTA heritability and genetic correlation in both univariate and bivariate GCTA analyses (http://spark.rstudio.com/ctgg/gctaPower/)^54^.

### Genotyping

The analysis is based on the genotypic data generated for 3,665 TEDS unrelated individuals by Wellcome Trust Sanger Institute, Hinxton, UK as part of the Wellcome Trust Case Consortium. Briefly, DNA was collected from 3,665 individuals using buccal swabs, which was thereafter genotyped using AffyrmetrixGeneChip 6.0 genotyping array. This yielded to genome-wide genotype calls for all individuals for around 600,000 SNPs (See Trzaskowski *et al.*, 2013 for full details)^55^. The data were thereafter imputed to 1000 genomes reference data using Impute 2 software and the standard quality control was applied. This left over 5.2 million SNPs available for molecular and GCTA analyses.

All analyses were carried out in accordance with the approved guidelines.

Ethical approval was received from King’s College London Ethics Committee: PNM/09/10-104 Twin Early Development Study; and informed consent was obtained from all subjects.

## Additional Information

**How to cite this article**: Rimfeld, K. *et al.* Pleiotropy across academic subjects at the end of compulsory education. *Sci. Rep.* **5**, 11713; doi: 10.1038/srep11713 (2015).

## Supplementary Material

Supplementary Information

## Figures and Tables

**Table 1:** GCSE grades and intelligence means and (standard deviations).

	**N**	**Whole sample**	**Male**	**Female**	**MZm**	**DZm**	**MZf**	**DZf**	**DZos**	**Sex**	**Zyg**	Sex*Zyg	**r**^**2**^
Intelligence	4,481	0 (0.99)	0.05(1.01)	(−0.03)(0.98)	0.002(.99)	0.06(1.03)	(−0.06)(0.98)	(−0.05)(.99)	0.003(0.99)	0.47	1.92	0.66	<0.01
English	12,099	8.91(1.21)	8.69(1.25)	9.10(1.16)	8.65(1.26)	8.74(1.22)	9.06(1.15)	9.08(1.18)	8.93(1.23)	169.7**	4.53**	0.06	0.03
Mathematics	12,013	8.94(1.45)	9.00(1.44)	8.89(1.34)	8.98(1.42)	9.04(1.43)	8.87(1.44)	8.89(1.46)	8.94(1.45)	5.79*	1.16	0.14	<0.01
Science	11,250	9.01(1.30)	9.01(1.29)	9.01(1.31)	9.01(1.28)	9.03(1.28)	9.00(1.29)	9.00(1.34)	9.01(1.30)	1.54	0.001	0.62	<0.01
Humanities	10,183	9.00(1.46)	8.80(1.48)	9.18(1.41)	8.75(1.52)	8.86(1.45)	9.15(1.40)	9.18(1.44)	8.99(1.45)	103.57**	4.23*	0.17	0.02
Second language	6,896	8.82(1.42)	8.65(1.46)	8.96(1.36)	8.61(1.47)	8.69(1.47)	8.92(1.36)	8.94(1.36)	8.83(1.41)	43.45**	2.86	0.01	0.01
Art	5,460	9.08(1.27)	8.86(1.32)	9.20(1.22)	8.85(1.29)	8.90(1.29)	9.16(1.25)	9.24(1.22)	9.08(1.27)	49.71**	0.88	0.89	0.02
Business informatics	4,661	8.96(1.26)	8.83(1.29)	9.09(1.22)	8.88(1.33)	8.86(1.29)	9.13(1.18)	9.03(1.21)	8.96(1.26)	25.52**	0.02	1.03	0.01

The maximum GCSE grade is 11 and the minimum grade is 4, representing grades A* to G. N = sample size after exclusions (individuals); MZ=monozygotic; DZ=dizygotic; m = male; f = female; os = opposite sex. ANOVA analyses were conducted after randomly selecting one twin per pair in order to test the main effect of sex and zygosity and the interaction between them. Results = F statistics, r^2^ = proportion of variance explained; *p < 0.05; **p < 0.01.

**Table 2:** Correlated factor solution for multivariate genetic analyses, showing phenotypic correlations (*r*_Ph_), genetic correlations (*r*_G_), shared-environmental (*r*_C_) and non-shared environmental (*r*_E_) correlations between intelligence and GCSE exam grades, with 95% confidence intervals in parentheses.

	**Intelligence**	**English**	**Mathematics**	**Science**	**Humanities**	**Second language**	**Art**	**Business informatics**
**r_Ph_**								
Intelligence	1							
English	0.52 (0.50–0.54)	1						
Mathematics	0.56 (0.53–0.58)	0.69 (0.69–0.70)	1					
Science	0.48 (0.46–0.51)	0.66 (0.65–0.67)	0.71 (0.70–0.72)	1				
Humanities	0.48 (0.45–0.50)	0.77 (0.76–0.78)	0.69 (0.68–0.70)	0.67 (0.66–0.69)	1			
Second language	0.48 (0.45–0.51)	0.71 (0.70–0.73)	0.67 (0.65–0.68)	0.63 (0.62–0.65)	0.68 (0.66–0.69)	1		
Art	0.36 (0.33–0.39)	0.57 (0.55–0.59)	0.50 (0.48–0.52)	0.49 (0.46–0.51)	0.57 (0.55–0.59)	0.53 (0.50–0.55)	1	
Business informatics	0.44 (0.40–0.47)	0.62 (0.60–0.63)	0.63 (0.61–0.64)	0.58 (0.56–0.60)	0.62 (0.60–0.63)	0.57 (0.55–0.60)	0.49 (0.46–0.51)	1
**r**_**G**_								
Intelligence	1							
English	0.65 (0.57–0.75)	1						
Mathematics	0.69 (0.62–0.76)	0.73 (0.70–0.75)	1					
Science	0.61 (0.51–0.73)	0.73 (0.69–0.78)	0.78 (0.75–0.82)	1				
Humanities	0.58 (0.48–0.69)	0.88 (0.84–0.92)	0.74 (0.71–0.78)	0.75 (0.70–0.80)	1			
Second language	0.59 (0.49–0.71)	0.83 (0.78–0.88)	0.72 (0.71–0.76)	0.68 (0.62–0.74)	0.76 (0.70–0.83)	1		
Art	0.44 (0.31–0.60)	0.65 (0.58–0.73)	0.56 (0.49–0.64)	0.51 (0.49–0.61)	0.62 (0.54–0.71)	0.56 (0.48–0.67)	1	
Business informatics	0.56 (0.44–0.72)	0.72 (0.64–0.81)	0.70 (0.63–0.77)	0.70 (0.60–0.80)	0.69 (0.61–0.78)	0.64 (0.54–0.75)	0.59 (0.43–0.75)	1
**r**_**C**_								
Intelligence	1							
English	0.79 (0.41–0.82)	1						
Mathematics	0.88 (0.51–1.00)	0.98 (0.93–1.00)	1					
Science	0.85 (0.41–1.00)	0.84 (0.74–0.93)	0.91 (0.84–0.91)	1				
Humanities	0.94 (0.54–0.94)	0.91 (0.84–0.97)	0.95 (0.90–1.00)	0.90 (0.80–0.98)	1			
Second language	0.93 (0.46–1.00)	0.83 (0.71–0.93)	0.88 (0.77–0.88)	0.89 (0.77–0.89)	0.90 (0.78–0.97)	1		
Art	0.82 (0.25–1.00)	0.81 (0.63–0.88)	0.83 (0.62–1.00)	0.87 (0.65–1.00)	0.91 (0.73–1.00)	0.91 (0.70–1.00)	1	
Business informatics	0.94 (0.40–1.00)	0.79 (0.79–0.96)	0.86 (0.71–0.90)	0.71 (0.54–0.71)	0.89 (0.73–1.00)	0.79 (0.58–0.96)	0.66 (0.35–0.98)	1
**r**_**E**_								
Intelligence	1							
English	0.19 (0.12–0.25)	1						
Mathematics	0.21 (0.19–0.27)	0.26 (0.22–0.30)	1					
Science	0.16 (0.08–0.23)	0.27 (0.23–0.31)	0.32 (0.28–0.36)	1				
Humanities	0.17 (0.09–0.24)	0.35 (0.31–0.39)	0.29 (0.25–0.33)	0.28 (0.23–0.32)	1			
Second language	0.15 (0.06–0.18)	0.23 (0.18–0.28)	0.31 (0.25–0.36)	0.26 (0.20–0.31)	0.22 (0.16–0.27)	1		
Art	0.10 (0.01–0.20)	0.18 (0.12–0.24)	0.10 (0.10–0.16)	0.14 (0.14–0.20)	0.20 (0.13–0.27)	0.12 (0.04–0.20)	1	
Business informatics	0.09 (–0.01–0.19)	0.21 (0.14–0.26)	0.27 (0.21–0.33)	0.23 (0.16–0.26)	0.23 (0.21–0.29)	0.24 (0.15–0.33)	0.17 (0.06–0.28)	1

**Table 3:** Correlated factor solution for multivariate genetic analyses, showing phenotypic correlations (*r*_Ph_), genetic correlations (*r*_G_), shared-environmental (*r*_C_) and non-shared environmental (*r*_E_) correlations between GCSE exam grades after correcting for intelligence, with 95% confidence intervals in parentheses.

	**English**	**Mathematics**	**Science**	**Humanities**	**Second language**	**Art**	**Business informatics**
**r_Ph_**							
English	1						
Mathematics	0.57 (0.54–0.59)	1					
Science	0.58 (0.55–0.60)	0.65 (0.63–0.67)	1				
Humanities	0.69 (0.67–0.71)	0.58 (0.55–0.60)	0.60 (0.58–0.62)	1			
Second language	0.62 (0.60–0.65)	0.55 (0.52–0.58)	0.55 (0.52–0.55)	0.57 (0.54–0.60)	1		
Art	0.47 (0.43–0.50)	0.40 (0.36–0.43)	0.39 (0.35–0.43)	0.48 (0.44–0.52)	0.40 (0.36–0.47)	1	
Business informatics	0.52 (0.48–0.55)	0.54 (0.50–0.57)	0.52 (0.49–0.56)	0.51 (0.48–0.55)	0.47 (0.42–0.51)	0.38 (0.32–0.43)	1
**r**_**G**_							
English	1						
Mathematics	0.54 (0.54–0.64)	1					
Science	0.64 (0.54–0.74)	0.69 (0.59–0.78)	1				
Humanities	0.81 (0.73–0.90)	0.57 (0.44–0.69)	0.68 (0.56–0.80)	1			
Second language	0.72 (0.59–0.85)	0.49 (0.31–0.65)	0.56 (0.40–0.71)	0.66 (0.51–0.83)	1		
Art	0.69 (0.52–0.89)	0.66 (0.44–0.90)	0.51 (0.30–0.73)	0.56 (0.37–0.75)	0.60 (0.34–0.88)	1	
Business informatics	0.62 (0.42–0.86)	0.57 (0.35–0.77)	0.63 (0.41–0.87)	0.65 (0.44–0.90)	0.58 (0.29–0.88)	0.65 (0.32–0.98)	1
**r**_**C**_							
English	1						
Mathematics	0.92 (0.72–0.94)	1					
Science	0.66 (0.31–0.94)	0.81 (0.62–0.95)	1				
Humanities	0.85 (0.59–0.99)	0.83 (0.61–0.98)	0.79 (0.52–1.00)	1			
Second language	0.81 (0.48–1.00)	0.81 (0.62–0.95)	0.75 (0.41–1.00)	0.8 (0.48–1.00)	1		
Art	0.27 (−0.39–0.72)	0.15 (−0.44–0.61)	0.40 (−0.20–0.86)	0.66 (0.15–0.93)	0.26 (−0.38–0.79)	1	
Business informatics	0.63 (0.06–1.00)	0.82 (0.38–1.00)	0.57 (−0.02–0.86)	0.65 (0.08–1.00)	0.6 (−0.04–1.00)	0.01 (−0.79–0.80)	1
**r**_**E**_							
English	1						
Mathematics	0.43 (0.37–0.48)	1					
Science	0.42 (0.36–0.48)	0.51 (0.45–0.56)	1				
Humanities	0.42 (0.35–0.48)	0.42 (0.35–0.48)	0.36 (0.29–0.43)	1			
Second language	0.37 (0.28–0.44)	0.45 (0.38–0.52)	0.39 (0.31–0.47)	0.28 (0.25–0.37)	1		
Art	0.20 (0.10–0.30)	0.17 (0.06–0.27)	0.21 (0.10–0.31)	0.26 (0.15–0.37)	0.20 (0.06–0.33)	1	
Business informatics	0.34 (0.23–0.43)	0.37 (0.27–0.47)	0.37 (0.26–0.46)	0.28 (0.17–0.39)	0.27 (0.14–0.40)	0.18 (0.01–0.35)	1

**Table 4:** Age-, sex-, and population stratification-adjusted univariate and bivariate genome-wide complex trait analysis (GCTA) for GCSE mathematics, science and English; N- number of individuals in the analyses; tr1- trait one; tr2- trait 2. Standard error in parentheses.

	**Additive genetic effects**			**Genetic correlation**	**Residual (non-genetic) effects**			Environmental (non-genetic residual) correlation*	**Variance_tr1**	**Variance_tr2**	**N_tr1/N_tr2**
	**Genetic variance_tr1**	**Genetic variance_tr2**	**Genetic covariance**		**Residual variance_tr1**	**Residual variance)_tr2**	**Residual covariance**				
Maths-Science	0.21 (0.11)	0.19 (0.12)	0.19 (0.09)	0.96 (0.16)	0.71 (0.11)	0.80 (0.12)	0.49 (0.10)	0.65 (0.06)	0.92 (0.03)	0.99 (0.03)	2502/2381
Maths-English	0.19 (0.11)	0.15 (0.10)	0.17 (0.09)	1.00 (0.19)	0.74 (0.11)	0.75 (0.11)	0.46 (0.09)	0.62 (0.06)	0.93 (0.03)	0.90 (0.03)	2502/2529
Science-English	0.17 (0.11)	0.15 (0.10)	0.16 (0.09)	1.00 (0.28)	0.81 (0.12)	0.75 (0.11)	0.41 (0.09)	0.53 (0.07)	0.97 (0.03)	0.90 (0.03)	2381/2529
English-Maths g regressed	0.13 (0.11)	0.14 (0.11)	0.14 (0.09)	1.00 (0.33)	0.81 (0.11)	0.78 (0.11)	0.41 (0.09)	0.52 (0.07)	0.94 (0.03)	0.93 (0.03)	2491/2458
English-Science g regressed	0.14 (0.11)	0.11 (0.11)	0.12 (0.09)	1.00 (0.49)	0.80 (0.11)	0.86 (0.12)	0.35 (0.09)	0.42 (0.08)	0.93 (0.03)	0.97 (0.03)	2491/2345
Maths-Science g regressed	0.19 (0.11)	0.15 (0.12)	0.17 (0.09)	1.00 (0.26)	0.74 (0.11)	0.83 (0.12)	0.44 (0.09)	0.57 (0.07)	0.93 (0.03)	0.97 (0.03)	2458/2345

*The current version of GCTA does not report the ‘environmental’ (i.e., non-genetic residual) correlation or its standard error. The environmental correlation (residual correlation) was derived here from the GCTA estimates using the following algorithm: C(e)_tr12/(√V(e)_tr1 × √V(e)_tr2), and the standard error was calculated using: Var(re) = re × re × (VarVe1/(4 × Ve1 × Ve1) + VarVe2/(4 × Ve2 × Ve2) + VarCe/(Ce× Ce) + CovVe1Ve2/(2 × Ve1 × Ve2 − CovVe1Ce/(Ve1 × Ce) − CovVe2Ce/(Ve2 × Ce)); SE(re) = sqrt[Var(re)], where re is the environmental correlation, Ve1 is the residual variance for trait 1, Ce is the residual covariance between two traits, VarVe1 is the sampling variance for Ve1 (residual variance for trait 1), VarCe is the sampling variance for Ce, CovVe1Ve2 is the sampling covariance between Ve1 and Ve2, and CovVe1Ce is the sampling covariance between Ve1 and Ce^26^.
