# Supplementary material for: Pleiotropy across academic subjects at the end of compulsory education
Source: Sci Rep. 2015 Jul 23;5:11713. doi: 10.1038/srep11713 (PMC4512149; doi:10.1038/srep11713)
Supplement: Supplementary Information [file srep11713-s1.pdf]

Pleiotropy across academic subjects at the end of compulsory education

Kaili Rimfeld, Yulia Kovas, Philip S. Dale, and Robert Plomin

Figures

Supplementary Figure S1. Multivariate model of additive genetic (A), shared environmental (C) and non-shared environmental (E) contributions to the covariance between traits. Two algebraically equivalent representations of the multivariate model are shown: (a) correlated factor solution of genetic correlation (*r*G), shared environmental correlation *(r*C) and non-shared environmental correlation (*r*E) and (b) Cholesky decomposition.

Tables

Supplementary Table S1. Model fitting results for univariate analyses for GCSE exam results and intelligence.

Supplementary Table S2. Model fitting results for univariate analyses for GCSE exam results after correcting for intelligence.

Supplementary Table S3. Multivariate model fitting results for Cholesky decomposition for GCSE exam grades and intelligence, genetic component of the variance.

- Correlated factor solution (a)

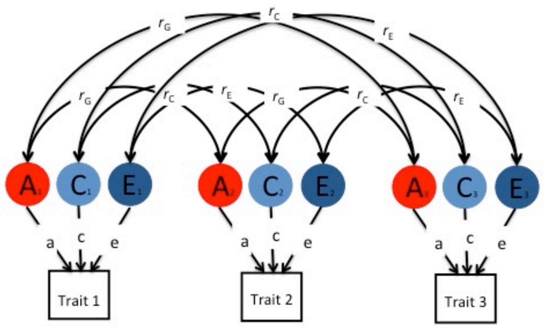

- Cholesky decomposition (b)

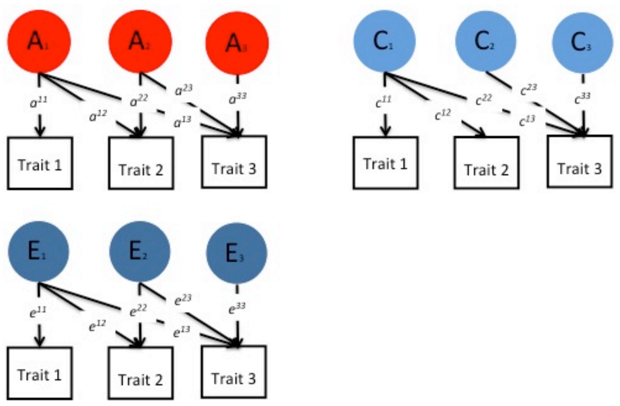

Supplementary Figure S1. Multivariate model of additive genetic (A), shared environmental (C) and non-shared environmental (E) contributions to the covariance between traits. Two algebraically equivalent representations of the multivariate model are shown: (a) correlated factor solution of genetic correlation (*r*G), shared environmental correlation *(r*C) and non-shared environmental correlation (*r*E) and b) Cholesky decomposition.

Supplementary Table S1. Model fitting results for univariate analyses for GCSE exam results and intelligence twin intraclass correlations, and additive genetic (A), shared environmental (C) and non-shared environmental (E) components of variance for GCSE language achievement (95% confidence intervals in parentheses). MZ=monozygotic twin pairs; DZ=dizygotic twin pairs, DZss= dizygotic same sax twin pairs; DSos=dizygotic opposite sex twin pairs

	Twin correlations						
	**MZ**	**DZ**	**DZss**	**DZos**	**A**	**C**	**E**
Intelligence	0.61 (0.57-0.66)	0.33 (0.28-0.38)	0.35 (0.28-0.42)	0.30 (0.22-0.38)	0.56 (0.43-0.65)	0.05 (0.01-0.16)	0.39 (0.35-0.43)
English	0.81 (0.80-0.83)	0.51 (0.48-0.53)	0.55 (0.51-0.58)	0.47 (0.44-0.51)	0.62 (0.58-0.67)	0.20 (0.15-0.24)	0.18 (0.17-0.19)
Mathematics	0.81 (0.80-0.83)	0.48 (0.46-0.51)	0.52 (0.48-0.55)	0.45 (0.41-0.48)	0.65 (0.60-0.70)	0.16 (0.11-0.21)	0.19 (0.17-0.20)
Science	0.76 (0.74-0.78	0.48 (0.45-0.50)	0.48 (0.44-0.52)	0.48 (0.43-0.51)	0.56 (0.51-0.62)	0.20 (0.15-0.25)	0.24 (0.22-0.25)
Humanities	0.75 (0.73-0.77)	0.48 (0.45-0.51)	0.52 (0.48-0.56)	0.44 (0.40-0.48)	0.54 (0.48-0.61)	0.21 (0.15-0.26)	0.25 (0.23-0.26)
Second language	0.76 (0.74-0.79)	0.48 (0.44-0.51)	0.53 (0.48-0.57)	0.43 (0.37-0.48)	0.56 (0.48-0.63)	0.20 (0.13-0.27)	0.24 0.22-0.26)
Art	0.72 (0.69-0.75)	0.44 (0.40-0.49)	0.51 (0.44-0.56)	0.38 (0.30-0.45)	0.54 (0.44-0.66)	0.18 (0.07-0.27)	0.28 (0.25-0.31)
Business informatics	0.70 (0.66-0.74)	0.41 (0.36-0.46)	0.49 (0.42-0.55)	0.33 (0.24-0.40)	0.56 (0.45-0.68)	0.14 (0.03-0.24)	0.30 (0.27-0.34)

Supplementary Table S2. Model fitting results for univariate analyses for GCSE exam results after correcting for intelligence, twin intraclass correlations, and additive genetic (A), shared environmental (C) and non-shared environmental (E) components of variance for GCSE language achievement (95% confidence intervals in parentheses). MZ=monozygotic twin pairs; DZ=dizygotic twin pairs, DZss= dizygotic same sax twin pairs; DSos=dizygotic opposite sex twin pairs

	Twin correlations						
	**MZ**	**DZ**	**DZss**	**DZos**	**A**	**C**	**E**
English	0.70 (0.65-0.73)	0.41 (0.35-0.45)	0.46 (0.40-0.52)	0.34 (0.26-0.41)	0.58 (0.47-0.70)	0.11 (0.01-0.21)	0.31 (0.27-0.34)
Mathematics	0.68 (0.64-0.71)	0.43 (0.38-0.48)	0.47 (0.40-0.53)	0.39 (0.31-0.46)	0.45 (0.33-0.56)	0.22 (0.12-0.31)	0.33 (0.30-0.37)
Science	0.69 (0.65-0.73)	0.42 (0.37-0.47)	0.40 (0.32-0.46)	0.45 (0.37-0.52)	0.51 (0.40-0.64)	0.15 (0.05-0.26)	0.34 (0.28-0.35)
Humanities	0.67 (0.63-0.72)	0.42 (0.36-0.48)	0.45 (0.37-0.52)	0.39 (0.30-0.47)	0.52 (0.39-0.65)	0.16 (0.04-0.27)	0.32 (0.28-0.36)
Second language	0.68 (0.65-0.73)	0.42 (0.35-0.49)	0.46 (0.35-0.54)	0.40 (0.30-0.50)	0.48 (0.32-0.64)	0.19 (0.05-0.33)	0.33 (0.28-0.38)
Art	0.67 (0.60-0.72)	0.42 (0.32-0.51)	0.51 (0.39-0.61)	0.29 (0.12-0.43)	0.50 (0.30-0.70)	0.17 (0.01-0.34)	0.33 (0.27-0.40)
Business informatics	0.61 (0.53-0.69)	0.34 (0.22-0.45)	0.37 (0.21-0.52)	0.31 (0.14-0.45)	0.49 (0.23-0.66)	0.11 (0.01-0.33)	0.40 (0.33-0.48)

Supplementary Table S3. Multivariate model fitting results for Cholesky decomposition for GCSE exam grades and intelligence, with 95% confidence intervals in the parenthesis, demonstrating the standardized squared paths for the genetic component of the variance.

	A1	A2	A3	A4	A5	A6	A7	A8
Intelligence	0.55 (0.45-0.61)							
English	0.26 (0.19-0.34)	0.34 (0.26-0.40)						
Mathematics	0.29 (0.23-0.38)	0.08 (0.03-0.13)	0.24 (0.19-0.26)					
Science	0.21 (0.14-0.30)	0.11 (0.05-0.16)	0.06 (0.02-0.08)	0.17 (0.14-0.21)				
Humanities	0.17 (0.18-0.25)	0.22 (0.15-0.29)	0.01 (0.01-0.03)	0.00 (0.00-0.01)	0.09 (0.05-0.12)			
Second language	0.20 (0.13-0.29)	0.20 (0.19-0.27)	0.01 (0.00-0.03)	0.00 (0.00-0.01)	0.00 (0.00-0.01)	0.14 (0.09-0.18)		
Art	0.10 (0.04-0.18)	0.12 (0.05-0.21)	0.01 (0.00-0.03)	0.00 (0.00-0.01)	0.00 (0.00-0.03)	0.00 (0.00-0.02)	0.29 (0.21-0.35)	
Business informatics	0.16 (0.09-0.26)	0.11 (0.09-0.19)	0.02 (0.00-0.05)	0.01 (0.00-0.03)	0.00 (0.00-0.02)	0.00 (0.00-0.02)	0.01 (0.00-0.04)	0.18 (0.09-0.24)
